# O-Acetylated Chemical Reporters of Glycosylation Can Display Metabolism-Dependent Background Labeling of Proteins but Are Generally Reliable Tools for the Identification of Glycoproteins

**DOI:** 10.3389/fchem.2020.00318

**Published:** 2020-04-28

**Authors:** Narek Darabedian, Bo Yang, Richie Ding, Giuliano Cutolo, Balyn W. Zaro, Christina M. Woo, Matthew R. Pratt

**Affiliations:** ^1^Department of Chemistry, University of Southern California, Los Angeles, CA, United States; ^2^Department of Chemistry and Chemical Biology, Harvard University, Cambridge, MA, United States; ^3^Biological Sciences, University of Southern California, Los Angeles, CA, United States; ^4^Department of Biological Science, University of Southern California, San Francisco, CA, United States

**Keywords:** bioorthogonal reporters, metabolic engineering, O-GlcNAc, proteomics, cysteine labeling

## Abstract

Monosaccharide analogs bearing bioorthogonal functionalities, or metabolic chemical reporters (MCRs) of glycosylation, have been used for approximately two decades for the visualization and identification of different glycoproteins. More recently, proteomics analyses have shown that per-*O*-acetylated MCRs can directly and chemically react with cysteine residues in lysates and potentially cells, drawing into question the physiological relevance of the labeling. Here, we report robust metabolism-dependent labeling by Ac_4_2AzMan but not the structurally similar Ac_4_4AzGal. However, the levels of background chemical-labeling of cell lysates by both reporters are low and identical. We then characterized Ac_4_2AzMan labeling and found that the vast majority of the labeling occurs on intracellular proteins but that this MCR is not converted to previously characterized reporters of intracellular O-GlcNAc modification. Additionally, we used isotope targeted glycoproteomics (IsoTaG) proteomics to show that essentially all of the Ac_4_2AzMan labeling is on cysteine residues. Given the implications this result has for the identification of intracellular O-GlcNAc modifications using MCRs, we then performed a meta-analysis of the potential O-GlcNAcylated proteins identified by different techniques. We found that many of the proteins identified by MCRs have also been found by other methods. Finally, we randomly selected four proteins that had only been identified as O-GlcNAcylated by MCRs and showed that half of them were indeed modified. Together, these data indicate that the selective metabolism of certain MCRs is responsible for S-glycosylation of proteins in the cytosol and nucleus. However, these results also show that MCRs are still good tools for unbiased identification of glycosylated proteins, as long as complementary methods are employed for confirmation.

## Introduction

**Figure 1:**
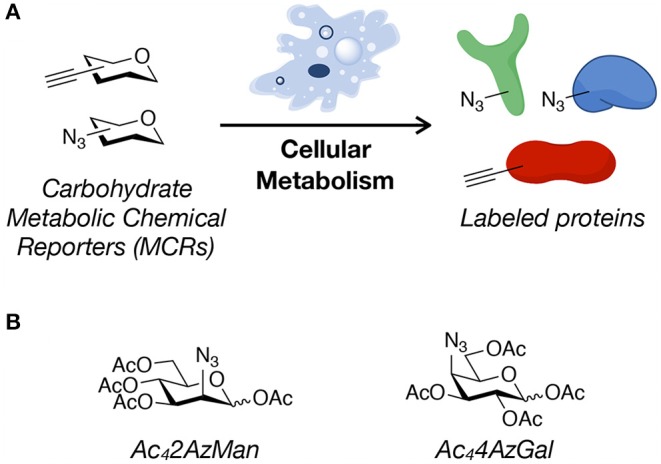
Metabolic chemical reporters. **(A)** Metabolic chemical reporters (MCRs) of protein glycosylation are traditionally per-O-acetylated, azide- or alkyne-bearing analogs of naturally-occurring monosaccharides. Upon treatment of living cells, MCRs can then be metabolized, resulting in protein labeling. **(B)** The structure of the potential MCRs studied here, 2-azido-2-deoxy-mannose (Ac_4_2AzMan) and 4-azido-4-deoxy-galactose (Ac_4_4AzGal).

Cellular biosynthetic pathways have been exploited for over two decades to incorporate chemical functionality into proteins and posttranslational modifications (Chuh et al., 2016; Gilormini et al., 2018; Parker and Pratt, 2020). For obvious biochemical reasons, metabolic probes or metabolic chemical reporters (MCRs) were traditionally designed to exploit known enzymatic promiscuities (Figure 1A). For example, the Bertozzi lab accomplished the first metabolic incorporation of reactive functionalities into complex carbohydrates by taking advantage of the enzymatic tolerances around the *N*-acetyl position of *N*-acetyl-mannosamine (Mahal et al., 1997; Saxon and Bertozzi, 2000) that had been previously discovered by Werner Reutter (Kayser et al., 1992). More specifically, small chemical-handles like azides or alkynes are tolerated at this position by the biosynthetic pathways that scavenge monosaccharides and convert them to the corresponding nucleotide sugar-donors. Glycosyltransferases can use these unnatural donors for the modification of proteins. A second bioorthogonal reaction step is then exploited to attached visualization or affinity tags for analysis. More recently, we and others have taken a broader approach to glycoprotein-MCR discovery through the synthesis and characterization of monosaccharide analogs that may not transit well-characterized biosynthetic pathways (Zaro et al., 2011, 2017; Chuh et al., 2014, 2017; Li et al., 2016; Shen et al., 2017; Darabedian et al., 2018). For instance, we demonstrated that 6-azido-6-deoxy-*N*-acetylglucosamine (6AzGlcNAc) can bypass the traditional GlcNAc-salvage pathway to generate uridine diphosphate sugar (UDP-6AzGlcNAc) (Chuh et al., 2014), resulting in labeling of O-GlcNAcylated proteins and suggesting that cellular metabolism is more accommodating to MCRs than previously appreciated. While this phenomenon was confirmed and expanded by ourselves and other labs, a recent analysis of per-*O*-acetylated MCRs by the Wang and Chen labs showed that they can chemically react with cysteines on proteins when incubated with cell lysates at moderate to high concentrations (0.2–2.0 mM) (Qin et al., 2018; Hao et al., 2019), raising concerns about how much reporter-labeling is due to enzymatic glycosylation.

Here, we analyze the “non-traditional” potential *O*-acetylated MCRs, 2-azido-2-deoxy-mannose (Ac_4_2AzMan) and 4-azido-4-deoxy-galactose (Ac_4_4AzGal) (Figure 1B). We find that treatment of mammalian cells with Ac_4_2AzMan results in robust labeling of proteins, while Ac_4_4AzGal does not result in any signal over background. In contrast to this live-cell labeling, we find that Ac_4_2AzMan and Ac_4_4AzGal display identical levels of chemical modification in cell lysates. We then further characterized Ac_4_2AzMan and found that the vast majority of the labeling is intracellular in nature with essentially no-detectable signal on the cell-surface. We and the Vocadlo lab independently showed that 2-azido-2-deoxy-glucose (2AzGlc) can be incorporated onto intracellular O-GlcNAc modification by the enzyme O-GlcNAc transferase (Shen et al., 2017; Zaro et al., 2017). These results raised the possibility that 2AzMan is converted to 2AzGlc by the enzyme *N*-acetylglucosamine 2-epimerase (Uniprot P51606); however, we used *in vitro* biochemistry to show that this is likely not the case. We do not know the mechanism by which Ac_4_2AzMan treatment labels proteins. However, comparison of this MCR to Ac_4_4AzGal indicates that direct cysteine-labeling by MCRs is not a universal property of per-*O*-acetylated monosaccharides. Instead, we believe that some background chemical labeling of proteins by MCRs is likely, but that it requires the conversion of the MCR into reactive species in cells. We believe that this background labeling pathway is distinct from glycosyltransferase-mediated modification of proteins. We then performed a meta-analysis of proteomic results from different strategies used to enrich and identify potentially O-GlcNAcylated proteins. We found that while MCRs gave the largest fraction of unique proteins, there was notable overlap with other techniques. Finally, we randomly chose four proteins that were only identified by an MCR as being O-GlcNAcylated and were able to confirm that two of them were indeed modified. Together, our results show that MCRs can result in chemical modification of intracellular proteins, but that this may be due to cellular metabolism of the reporter instead of direct reaction of the per-O-acetylated monosaccharide with cysteines. However, we also show that MCRs are still powerful discovery tools that should be used in conjunction with complementary techniques to confirm the glycosylation status of any identified protein.

## Materials and Methods

### General Information

Reagents and solvents were obtained from various commercial suppliers and were used without further purification. Thin-layer chromatography (TLC) was performed on EMD Silica Gel 60 F_254_ plates and visualized by ceric ammonium molybdate or UV. Flash chromatography was perfumed on 60 Ã silica gel (EMD. ^1^H spectra were obtained on a 400 MHz on a Varian spectrometer Mercury 400 and chemical shifts are recorded in ppm (δ) relative to solvent and coupling constants (J) are reported in Hz.

### Synthesis of Ac_4_4AzGal

1,2,3,6-Tetra-*O*-acetyl-D-glucopyranose (250 mg, 0.72 mmol) was dissolved in 7 mL CH_2_Cl_2_ and 1 mL of pyridine was added and cooled to 0°C. Triflic anhydride (0.49 mL, 2.87 mmol) was then added dropwise and the reaction was allowed to stir at 0°C for 1 h. The mixture was then diluted with CH_2_Cl_2_ and washed twice with 1 M hydrogen chloride, once with saturated sodium bicarbonate, and once with brine. The organic layer was dried over sodium sulfate, filtered, and concentrated. The resulting oil was dissolved in 5 mL DMF, NaN_3_ (234 mg, 3.6 mmol) was added, and the mixture was stirred for 1.5 h. The reaction was then diluted with ethyl acetate and the organic layer was washed twice with water and once with brine. The organic layer was dried over sodium sulfate, filtered, and concentrated. The product was then purified by column chromatography (10–30% acetone in hexanes, 3 steps) to afford 205 mg of the product. ^1^H NMR (400 MHz, Chloroform-*d*) δ 6.31 (t, *J* = 1.6 Hz, 1H), 5.41–5.39 (m, 2H), 4.24–4.16 (m, 4H), 2.15 (s, 3H), 2.14 (s, 3H), 2.09 (s, 3H), 2.03 (s, 3H). These data are consistent with previously published characterization (Chen and Withers, 2018).

### Cell Culture

H1299 cells were grown in RPMI media (GenClone), while HeLa cells were grown in DMEM high glucose media (GenClone). In both cases, media was supplemented with 10% Fetal Bovine Serum (Altanta Biologicals). NIH3T3 cells were grown in DMEM high glucose (GenClone) supplemented with 10% Fetal Calf Serum (Altanta Biologicals). All Cell lines were grown at 37°C and 5.0% CO_2_.

### Methanol/Chloroform/H_2_O Precipitation

Proteins were recovered through first addition of a 3 × volume of methanol, a 0.75 × volume of chloroform, and a 2 × volume of H_2_O. The resulting mixtures were then subjected to mixing by vortexing and centrifugation (5 min, 5,000 × g). The aqueous phase separates at the top of the mixture and was removed and discarded without disturbing the interface layer. An additional 2.5 × volume of methanol was then added, followed by mixing by vortexing, and pelleting of protein by centrifugation (10 min, 5,000 × g).

### Background Lysate-Labeling

HeLa cells were collected by trypsinization and washed two times with PBS (2 min, 2,000 × g, 4°C).

#### Native Lysis Conditions

The resulting cell-pellets were resuspended in 400 μL of PBS with 5 mg/mL Protease Inhibitor and then tip sonicated in the ice for 45 s (15 s on, 10 s off). Protein concentration was normalized using a BCA assay (Pierce, ThermoScientific) and diluted to 2 mg mL^−1^. To 100 μL (200 μg) of this protein solution, was added either Ac_4_2AzMan or Ac_4_4AzGal from either a 2 or 10 mM stock solution in DMSO to give a final concentration of 200 μM or 2 mM, respectively. After incubation of this mixture at 37°C for 2 h, the lysates were precipitated by the addition of 800 μL of cold MeOH and incubation at −80° for 1 h. The precipitates were collected by centrifugation at 8,000 × g, 5 min at 4°C and washed twice with cold MeOH. The supernatant was removed, and the pellet was allowed to air-dry, and then 188 μL 1% SDS buffer (1% SDS, 150 mM NaCl, 50 mM triethanolamine pH 7.4) was added to each sample. The mixture was sonicated in a bath sonicator to ensure complete dissolution. The resulting protein mixture was then subjected to the CuAAC conditions described below.

#### Denaturing Lysis Conditions

The resulting cell-pellets were resuspended in 100 μL 1% SDS with 5 mg/mL Protease Inhibitor and then tip sonicated for 15 s. Protein concentration was normalized using a BCA assay (Pierce, ThermoScientific) and diluted to 2 mg mL^−1^. To 50 uL (100 μg) of this protein solution, was added either Ac_4_2AzMan or Ac_4_4AzGal from either a 2 or 10 mM stock solution in DMSO to give a final concentration of 200 μM or 2 mM, respectively. After incubation of this mixture at 37°C for 2 h, the reaction was diluted with 50 μL of 4% SDS (4% SDS, 50 mM triethanolamine pH 7.4, 150 mM NaCl) and subjected to methanol/chloroform/H_2_O precipitation. The supernatant was removed, and the pellet was allowed to air-dry, and then 94 μL 1% SDS buffer (1% SDS, 150 mM NaCl, 50 mM 50 mM triethanolamine pH 7.4) was added to each sample. The mixture was sonicated in a bath sonicator to ensure complete dissolution. The resulting mixture was then subjected to the CuAAC conditions described below.

#### CuAAC

To 100 μg of the protein mixtures above was added fresh click chemistry cocktail (6 μL)[alkyne-TAMRA tag (Click Chemistry Tools, 100 μM, 10 mM stock solution in DMSO), tris(2-carboxyethyl)phosphine hydrochloride (TCEP) (1 mM, 50 mM freshly prepared stock solution in water), tris[(1-benzyl-1-*H*-1,2,3-triazol-4-yl)methyl]amine (TBTA) (Click Chemistry Tools, 100 μM, 10 mM stock solution in DMSO), CuSO_4_·5H2O (1 mM, 50 mM freshly prepared stock solution in water)]. After 1 h, the CuAAC reaction was subjected to methanol/chloroform/H_2_O precipitation. The resulting protein-pellet was air-dried (5–10 min), and then 25 μL of 4% SDS buffer (4% SDS, 150 mM NaCl, 50 mM 50 mM triethanolamine pH 7.4) was added to each sample. The mixture was sonicated in a bath sonicator until all the protein was dissolved, and 25 μL of 2 × SDS-free loading buffer (20% glycerol, 0.2% bromophenol blue, 1.4% β-mercaptoethanol, pH 6.8) was then added. The samples were boiled for 5 min at 97°C, and 40 μg of protein was then separated by SDS-PAGE. The resulting gels were then visualized using a Typhoon 9400 Variable Mode Imager (GE Healthcare) with a 532 nm laser for excitation and a 30 nm bandpass filter centered at 610 nm for detection.

### Metabolic Labeling

To cells at 80–85% confluency, media was exchanged for fresh media containing Ac_4_2AzMan, Ac_4_4AzGal, Ac_3_6AzGlcNAc, Ac_4_ManNAz, Ac_4_5SGlcNAc (1,000 × stock in DMSO), or DMSO vehicle as indicated. Cells were removed from the plate by trypsinization, collected by centrifugation (2 min, 2,500 × g), and gently washed with PBS (1 mL, 2 ×). The resulting pellets were lysed in 100 μL of 1% NP-40 lysis buffer [1% NP-40, 150 mM NaCl, 50 mM triethanolamine (TEA) pH 7.4] with complete, Mini, EDTA-free Protease Inhibitor Cocktail Tablets (Sigma Aldrich) for 20 min. Any remaining cell debris was then removed by centrifugation (10 min, 10, 000 × g, 4°C). The soluble fraction, or soluble cell lysate, was collected and a BCA assay (Pierce, ThermoScientific) was used to determine protein concentration, which normalized to 1 μg μL^−1^ using more lysis buffer. CuAAC was then performed on 200 μg of protein by adding 12 μL of fresh click chemistry cocktail [alkyne-TAMRA tag (Click Chemistry Tools, 100 μM, 10 mM stock solution in DMSO), tris(2-carboxyethyl)phosphine hydrochloride (TCEP) (1 mM, 50 mM freshly prepared stock solution in water), tris[(1-benzyl-1-H-1,2,3-triazol-4-yl)methyl]amine (TBTA) (Click Chemistry Tools, 100 μM, 10 mM stock solution in DMSO), CuSO_4_·5H_2_O (1 mM, 50 mM freshly prepared stock solution in water)]. The reaction was gently vortexed before proceeding for 1 h at room temperature. After this time, ice cold methanol (1 mL) was then added to the reaction, and the samples were incubated at −20°C for 2 h. The samples were then centrifuged (10 min, 10,000 × g, 4°C), resulting in a pellet of the proteins. This protein-pellet was air-dried (5–10 min), and 25 μL of 4% SDS buffer (4% SDS, 150 mM NaCl, 50 mM 50 mM triethanolamine pH 7.4) was then added. The mixture was completely dissolved using a bath sonicator, followed by addition of 25 μL of 2 × SDS-free loading buffer (20% glycerol, 0.2% bromophenol blue, 1.4% β-mercaptoethanol, pH 6.8). The samples were fully denatured by incubation at 97°C for 5 min, and 40 μg of protein was then separated by SDS-PAGE. The resulting gels were then visualized using a Typhoon 9400 Variable Mode Imager (GE Healthcare) with a 532 nm laser for excitation and a 30 nm bandpass filter centered at 610 nm for detection.

### β-Elimination

β-Elimination was performed as previously described (Darabedian et al., 2018).

### PNGase F Treatment

PNGase F was obtained from New England Biolabs and treatment was performed according to the manufacturer's protocol with some changes as previously described (Darabedian et al., 2018).

### Flow Cytometry of Cell-Surface Labeling With DBCO-Biotin

NIH3T3 cells were grown in 10 cm plates at 80–85% confluency and treated with 200 μM MCRs or Ac_4_GlcNAc in triplicate for 16 h. The media was then removed and cells were washed with DPBS before being detached from the plate by incubation in 10 mL of 10 mM EDTA in DPBS at 37°C for 10 min. Cells were collected by centrifugation (5 min, 800 × g, 4°C) and were washed three times with DPBS (5 min, 800 × g, 4°C). Cells were then resuspended in 200 μL PBS containing DBCO-biotin (Click Chemistry Tools, 60 μM, 10 mM stock in DMSO) for 1 h at RT, after which time they were washed three times with DPBS (5 min, 800 g at 4°C) before being resuspended in ice-cold PBS containing fluorescein isothiocynate (FITC) conjugated avidin (Sigma, 5 μg μL^−1^, 1 mg/mL stock) for 30 min at 4 °C. Cells were then washed three times in DPBS (5 min, 800 × g, 4°C) and then resuspended in 400 μL PBS containing propidium iodide (2.5 μg mL^−1^ in DPBS, 1 mg/mL stock in DPBS] for 30 min. A total of 50,000 cells were analyzed on a BD Accuri C6 Flow Cyometer, and dead cells (propidium iodide positive) were excluded.

### GlcNAc-2-Epimerase Cloning and Expression

Condon optimized GlcNAc-2-Epimerase (11–427) was purchased from Integrated Device Technology and cloned into pET-28b vector between NdeI and HindIII using standard molecular cloning techniques. Plasmid is available upon request. Pet28b-6XHis-GlcNAc-2-Epimerase (11–427) was subsequently transformed into BL21 *E. coli* (Novagen). Terrific broth (1.8 L) containing kanamycin (50 μg mL^−1^) was inoculated with 24 mL of starter culture grown overnight at 37°C. The culture was grown at 37°C until an OD (A600) of 0.80 was obtained, at which time expression was induced with addition of 1 mM isopropyl β-D-1-thiogalacto-pyranoside (IPTG) for 5 h at 37°C. Cells were harvested by centrifugation (10 min, 6,000 × g, 4°C). The pooled pellets were suspended in 20 mL Buffer A (250 mM NaH_2_PO_4_, 300 mM NaCl, 20 mM imidazole, pH 7.4) and tip sonicated for 12 min (30 s on, 15 s off). The resulting lysate was then centrifuged (15 min, 30,000 × g, 4°C), and the supernatant was transferred into a new tube and centrifuged again (30 min, 30,000 × g, 4°C). The supernatant was transferred into a new tube and 2 mL of pre-washed Cobalt Metal Affinity Resin (Genesee Scientific) was added and placed onto a rotator for 1 h at 4°C. The solution was then transferred to a gravity-flow column, allowed to drain, and the beads were washed with an additional 100 mL of Buffer A. 6XHis-GlcNAc-2-Epimerase (11–427) was eluted with 5 mL fractions of Buffer B (25 mM NaH_2_PO_4_, 300 mM NaCl, 250 mM imidazole, pH 7.4). Fractions containing 6XHis-GlcNAc-2-Epimerase (11–427) were concentrated to 2 mL using a 10 kDa cutoff Amicon Ultra-15 Centrifugal Filter. The concentrate was dialyzed overnight into 2 L of dialysis buffer (20 mM NaH_2_PO_4_, 1 mM EDTA, 5% glycerol, 0.5% 2-Mercaptoethanol, pH 7.0). The dialyzed solution was then concentrated to 0.5 mL using a 10 kDa cutoff Amicon Ultra-15 Centrifugal Filter and stored at −80°C in single use aliquots.

### HPLC Analysis for GlcNAc-2-Epimerase Conversion

To a 500 μL microcentrifuge tube was added 10 μL of phosphate buffer (1 M, pH 7.5), 10 μL of ATP (50 mM, dissolved in water), 10 μL of MgCl_2_ (100 mM, dissolved in water), 20 μL of ManNAc or 2AzMan (500 mM, dissolved in water), and 5 μL of GlcNAc-2-Epimerase (or water). Water, 45 μL for samples containing GlcNAc-2-Epimerase or 50 μL for null samples, was then added to the reaction mixtures. The samples were then incubated at 37°C for 12 h and then lyophilized. The resulting solids were suspended in 75 μL of pyridine and then 25 μL of acetic anhydride was added and allow to rotate for 16 h. Then 5 μL of each solution was diluted to 100 μL using water and 20 μL was injected onto an Agilent Eclipse XDB-C18 (5 μm, 4.6 × 150 mm) running at 1 ml/min and PDA set to a wavelength 200 nm. Buffer A was H_2_O containing 0.1% TFA, buffer B was 90% ACN, 10% H_2_O containing 0.1% TFA. HPLC conditions for 2AzMan were 10 min at 25% B and then a ramp to 70% over 20 min. HPLC conditions for ManNAc were 10 min at 10% B and then a ramp to 50% over 20 min.

### Biotin Enrichment for Proteomics

H1299 cells were treated as indicated and cells were collected by trypsinization and pelleted by centrifugation for (2 min, 2,000 × g), followed by washing 2 × with PBS. The resulting cell-pellets were resuspended in 4% SDS buffer (4% SDS, 10 mM TEA pH 7.4, 150 mM NaCl) containing c0mplete, Mini, EDTA-free Protease Inhibitor Cocktail Tablets (Roche), tip sonicated for 15 s, and cleared by centrifugation (10 min, 10,000 × g, 15°C). Soluble protein concentration was normalized by BCA assay (Pierce, ThermoScientific) to 1 mg mL^−1^, and 1 mg of total protein was subjected to the appropriate amount of click chemistry cocktail containing alkyne-biotin (Click Chemistry Tools) for 1 h, then 10 μL of 0.5 M EDTA was added. Then proteins were precipitated by adding a 7.5 mL of methanol, 1.9 mL of CHCl_3_, and 5 mL of H_2_O followed by vortexing and centrifugation (5 min, 5,000 × g). The aqueous phase was discarded without disturbing the interface layer after which 3.7 mL of methanol was added, vortexed, and centrifuged (10 min, 10,000 × g, 4°C). The supernatant was removed and the pellet was allowed to air-dry for 5 min and then a 100 μL of 4% SDS was added. The mixture was sonicated in a bath sonicator to ensure complete dissolution, and 1.9 mL of 10 mM TEA, pH 7.4, 150 mM NaCl was added followed by 125 μL of high-capacity NeutrAvidin beads (ThermoScientific, pre-washed three times with 0.2% SDS, 150 mM NaCl, 50 mM TEA pH 7.4) and incubated on a rotator for 1.5 h. Afterwards, the beads were washed with 6 × 1% SDS in PBS, 3 × 4M urea in PBS, and 8 × 50 mM NH_4_HCO_3_. The beads were then resuspended in 1 mL of 50 mM NH_4_HCO_3_, 10 mM TCEP (pH 8) and incubated for 30 min with gentle shaking. Afterwards, the resin was washed with 50 mM NH_4_HCO_3_ and the beads were resuspended in 1 mL of 10 mM iodoacetamide (pH 8) in 50 mM NH_4_HCO_3_ and incubated in the dark for 30 min. The beads were then washed 3 × 50 mM NH_4_HCO_3_, and resuspended in 100 μL 50 mM NH_4_HCO_3_. Then 2 μL of CaCl_2_ (200 mM in H2O) and 2 μL of trypsin (Sequencing Grade, Promega, 0.1 μg/μl) were added and incubated for 18 h at 37°C. The beads were centrifuged, the supernatants were transferred into clean tubes, and the beads were washed with an additional 100 μL 1% formic acid, 100 μL 15 % acetonitrile in H_2_O and 100 μL 1% FA in H_2_O. The combined elution and wash were desalted on C18 Spin Columns (Pierce, ThermoScientific) according to the manufacturer's protocol and lyophilized to dryness.

### Proteomics

A nanoElute was attached in line to a timsTOF Pro equipped with a CaptiveSpray Source (Bruker). Chromatography was conducted at 40°C through a 25 cm reversed-phase Aurora Series C18 column (IonOpticks) at a constant flow-rate of 0.4 μL/min. Mobile phase A was 98/2/0.1% Water/acetonitrile/formic acid (v/v/v) and phase B was acetonitrile with 0.1% Formic Acid (v/v). During a 120 min method, peptides were separated by a 4-step linear gradient (0% to 15% B over 60 min, 15% to 23% B over 30 min, 23% to 35% B over 10 min, 35% to 80% over 10 min) followed by a 10 min isocratic flush at 80% for 10 min before washing and a return to low organic conditions. Experiments were run as data-dependent acquisitions with ion mobility activated in parallel accumulation serial fragmentation (PASEF) mode. MS and MS/MS spectra were collected with *m*/*z* 400–1,500 and ions with *z* = +1 were excluded. Raw data files were processed with Peaks Studio. Fixed modifications included +57.02146 C. Variable modifications included Acetyl +42.010565 N-term, pyro-Glu −17.026549 N-term Q, pyro-Glu −18.010565 N-term E. Precursor tolerance 30.0 ppm. False discovery rate was set to 0.01 with significance calculated using ANOVA.

### Chemical Enrichment of Glycoproteins and Sample Preparation for IsoTag

H1299 cells were treated as indicated and cells were collected by trypsinization and pelleted by centrifugation for (2 min, 2,000 × g), followed by washing 2 × with PBS. The resulting cell-pellets were lysed on ice by probe tip sonication in 1 × PBS + 1% SDS (1 mL), containing EDTA-free Pierce Halt^TM^ protease inhibitor cocktail. Debris were removed from the cellular lysate by centrifugation (20,000 × g) for 20 min at 4°C and the supernatant transferred to a new Eppendorf tube. A BCA protein assay (Pierce) was performed and protein concentration was adjusted to 3.5 μg/μL with lysis buffer. Protein lysate (1.4 mg, 400 μL) was treated with a pre-mixed solution of the click chemistry reagents [100 μL; final concentration of 200 μM IsoTaG silane probe (3:1 heavy:light mixture), 500 μM CuSO_4_, 100 μM THPTA, 2.5 mM sodium ascorbate] and the reaction was incubated for 3.5 h at 24°C. The click reaction was quenched by a methanol-chloroform protein precipitation [aqueous phase/methanol/chloroform = 4:4:1 (v/v/v)]. The protein pellet was allowed to air dry for 5 min at 24°C. The dried pellet was resuspended in 1 × PBS + 1% SDS (400 μL) by probe tip sonication and then diluted in PBS (1.6 mL) to a final concentration of 0.2% SDS. Streptavidin-agarose resin [400 μL, washed with PBS (3 × 1 mL)] were added to the protein solution and the resulting mixture was incubated for 12 h at 24°C with rotation. The beads were washed using spin columns with 8 M urea (5 × 1 mL), and PBS (5 × 1 mL). The washed beads were resuspended in 500 μL PBS containing 10 mM DTT and incubated at 37°C for 30 min, followed by addition of 20 mM iodoacetamide for 30 min at 37°C in the dark. The reduced and alkylated beads were collected by centrifugation (1,500 × g) and resuspended in 520 μL PBS. Urea (8 M, 32 μL) and trypsin (1.5 μg) was added to the resuspended beads and digestion was performed for 16 h at 37°C with rotation. The beads were washed three times with PBS (200 μL) and distilled water (200 μL). The IsoTaG silane probe was cleaved with 2% formic acid/water (2 × 200 μL) for 30 min at 24°C with rotation and the eluent was collected. The beads were washed with 50% acetonitrile-water + 1% formic acid (2 × 500 μL), and the washes were combined with the eluent to form the cleavage fraction. The cleavage fractions were dried in a vacuum centrifuge and stored at −20°C until analysis.

### Mass Spectrometry Parameters Used for Glycoproteomics

A Waters nanoAcquity system was coupled to a ThermoScientific Orbitrap Fusion Tribrid with a nano-electrospray ion source. Half of the sample was reconstituted in 10 μL of 5% acetonitrile and 0.1% formic acid in water, loaded onto a C18 trap column (WATERS Cat # 186008821 nanoEase MZ Symmetry C18 Trap Column, 100 Å, 5 μm × 180 μm × 20 mm), and separated on an analytical column (WATERS Cat # 186008795 nanoEase MZ Peptide BEH C18 Column, 130 Å, 1.7 μm × 75 μm × 250 mm). Mobile phases A and B were water with 0.1% formic acid (v/v) and acetonitrile with 0.1% formic acid (v/v), respectively. Peptides were separated with a linear gradient from 5 to 30% B within 95 min, followed by an increase to 50% B within 15 min and further to 98% B within 10 min, and re-equilibration. The instrument parameters were set as previously described (Ramirez et al., 2020) with minor modifications. Briefly, MS1 spectra were recorded from m/z 400–2,000 Da. If oxonium product ions (HexAz0Si +288.1190 Da; HexAz2Si +290.1316 Da) were observed in the HCD spectra, ETD with supplemental activation (35%) was performed in a subsequent scan on the same precursor ion selected for HCD. The raw data was processed using Proteome Discoverer 2.4 (Thermo Fisher Scientific). Both HCD and EThcD spectra were searched against a database containing the Swissprot 2018 annotated human proteome (20,355 proteins, downloaded on Feb. 21, 2019) and contaminant proteins using Sequest HT and Byonic algorithms. The searches were performed with the following guidelines: trypsin as enzyme, 2 missed cleavages allowed; 10 ppm mass error tolerance on precursor ions; 0.02 Da mass error tolerance on fragment ions; variable modifications (methionine oxidation, +15.995 Da; carbamidomethyl cysteine, +57.021 Da; and others as described below). Intact glycopeptide searches allowed for the tagged hexose, or for the mono-acetylated, di-acetylated or tri-acetylated form (HexAz0Si, +287.112 Da; HexAz2Si, +289.124 Da; mono-acetylated HexAz0Si, +329.122 Da; mono-acetylated HexAz2Si, + 331.135 Da; di-acetylated HexAz0Si, +371.133 Da; di-acetylated HexAz2Si, +373.145 Da; tri-acetylated HexAz0Si, +413.143 Da; and tri-acetylated HexAz2Si, +415.156 Da) on asparagine, cysteine, serine, and threonine. Glycopeptide spectral assignments passing a false discovery rate of 1% at the spectrum level based on a target decoy database were manually validated for an isotope precursor pattern.

### UDP-GalNAz Chemoenzymatic Labeling and Cu(I)-Catalyzed [3 + 2] Azide–Alkyne Cycloaddition (CuAAC) for Western Blotting

H1299 cells were grown to 80% confluency, collected by trypsinization, and washed two times with PBS with collection by centrifugation (2 min, 2,000 × g, 4°C). The cell pellets were then resuspended in 2.5 mL 4% SDS (4% SDS, 50 mM TEA, 150 mM NaCl, pH 7.4) containing 12.5 mg of c0mplete Mini Protease Inhibitor Cocktail (Roche). The suspended cells were subjected to tip sonication (3 × 10 s on, 10 s off) followed by centrifugation (10 min, 10,000, 15°C). The soluble protein was collected and the concentration was normalized by BCA assay (Pierce) to 1 mg mL^−1^ using 1% SDS (1% SDS, 50 mM TEA, 150 mM NaCl, pH 7.4). Proteins were subjected to methanol/chloroform/H_2_O precipitation. The resulting protein pellet was allowed to air-dry for 5–10 min before being resuspended in 10% of the original volume using 1% SDS chemoenzymatic buffer (1% SDS, 20 mM HEPES, pH 7.9). Protein concentration was normalized using the BCA Assay and diluted to 2.5 mg mL^−1^ in 1% SDS chemoenzymatic buffer. To 2,400 μL of resuspended protein (6 mg) was added 2,940 μL of H_2_O, 4,800 μL of labeling buffer (2.5 ×; 5% IGEPAL CA-630, 125 mM NaCl, 50 mM HEPES, pH 7.9), 660 μL of MnCl_2_ (100 mM in H_2_O), and 900 μL of UDP-GalNAz (0.5 mM in 10 mM HEPES, pH 7.9) and vortexed. Finally, 300 μL of purified GalT Y289L or H_2_O was added, and the reaction mixture was incubated for 20 h at 4°C. The unreacted UDP-GalNAz was then removed by methanol/chloroform/H_2_O precipitation. Air-dried protein pellets were resuspended in 1,500 μL of 4% SDS. To generate inputs, 40 μL was removed and combined with 40 μL of 2 × loading buffer (20% glycerol, 0.2% bromophenol blue, pH 6.8, and 14 μL/mL β-mercaptoethanol). The remaining labeled lysate was diluted to 6 mL using SDS-free buffer (150 mM NaCl, 50 mM TEA pH 7.4) and newly made click chemistry cocktail (420 μL) was added to each sample [alkyne-azo-biotin (Click Chemistry Tools, 100 μM, 5 mM stock solution in DMSO); tris(2-carboxyethyl)phosphine hydrochloride (TCEP) (1 mM, 50 mM freshly prepared stock solution in water); tris[(1-benzyl-1-H-1,2,3- triazol-4-yl)methyl]amine (TBTA) (Click Chemistry Tools, 100 μM, 10 mM stock solution in DMSO); CuSO_4_·5H_2_O (1 mM, 50 mM freshly prepared stock solution in water). After 1 h, 60 μL of 0.5 M EDTA was added, and then the proteins were subjected to methanol/chloroform/H_2_O precipitation. The resulting proteins were then suspended in 600 μL of 4% SDS buffer.

### Metabolic Chemical Labeling and Cu(I)-Catalyzed [3 + 2] Azide–Alkyne Cycloaddition (CuAAC) for Western Blotting

H1299 cells were grown to 80% confluency, collected by trypsinization, and washed two times with PBS with collection by centrifugation (2 min, 2,000 × g, 4°C). The cell pellets were then resuspended in 1 mL 4% SDS containing 5 mg of c0mplete Mini Protease Inhibitor Cocktail (Roche). The suspended cells were subjected to tip sonication (3 × 10 s on, 10 s off) and followed by centrifugation (10 min, 10,000, 15°C). The soluble protein was collected and the concentration was normalized by BCA assay (Pierce) to 4 μg μL^−1^ in 4% SDS buffer. To generate inputs, 40 μL was removed and combined with 40 μL of 2 × loading buffer. To 1,500 μL of lysate (6 mg of protein) was added 780 μL of 1.25 × SDS-buffer (1.25% SDS, 50 mM TEA, 150 mM NaCl, pH 7.4), 3.3 ml of SDS-free buffer, and 420 μL of freshly made click chemistry cocktail was added [alkyne-azo-biotin (Click Chemistry Tools, 100 μM, 5 mM stock solution in DMSO); tris(2-carboxyethyl)phosphine hydrochloride (TCEP) (1 mM, 50 mM freshly prepared stock solution in water); tris[(1-benzyl-1-H-1,2,3- triazol-4-yl)methyl]amine (TBTA) (Click Chemistry Tools, 100 μM, 10 mM stock solution in DMSO); CuSO_4_·5H_2_O (1 mM, 50 mM freshly prepared stock solution in water). After 1 h, the proteins were subjected to methanol/chloroform/H_2_O precipitation. The resulting protein pellets were then suspended in 600 uL of 4% SDS buffer.

### Biotin Enrichment and Western Blotting

To the labeled proteins (600 μL, 6 mg) was added 11.4 mL of SDS-free buffer and 300 μL of high-capacity NeutrAvidin beads (ThermoScientific, pre-washed three times with 0.2% SDS, 150 mM NaCl, 50 mM TEA pH 7.4), then the mixture was then incubated for 1.5 h. The resulting mixture was transferred into a gravity flow chromatography column and drained. The beads were washed 10 × with 3 mL of 1% SDS in PBS and then transferred into a dolphin nose tube. Each sample was then incubated with 300 μL of 25 mM sodium hydrosulfite for 30 min, beads were then collected by centrifugation (2 min, 2,500 × g) and the supernatant was collected. The procedure was repeated two more times. The supernatant was pooled, 4 × volume of ice-cold methanol was added, and was placed at −20°C for 2 h. Precipitated proteins were then collected by centrifugation (10 min, 10,000 × g, 4°C). The supernatant was removed and the pellet was allowed to air-dry for 10 min and then 37.5 μL of 4% SDS buffer was added to each sample. The mixture was sonicated in a bath sonicator to ensure complete dissolution and then 37.5 μL of 2X SDS-free loading buffer was added. The samples were boiled for 5 min at 97°C and 20 μL of input or 25 μL of enriched sample was loaded per lane for SDS-PAGE separation.

## Results

**Figure 2:**
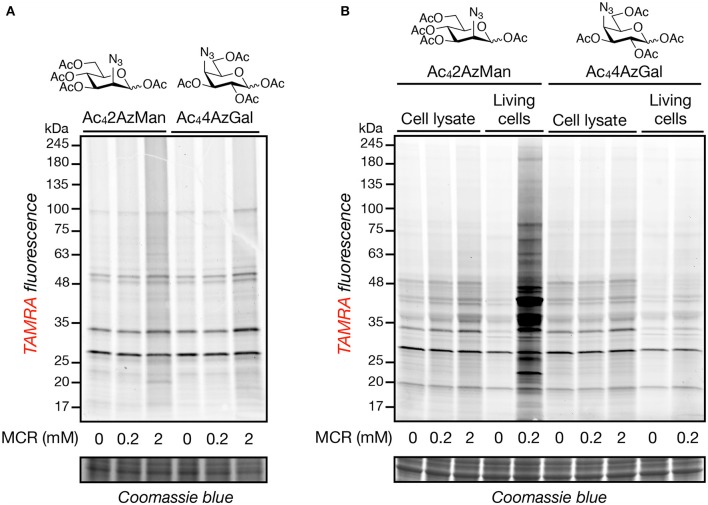
Neither Ac_4_2AzMan nor Ac_4_4AzGal chemically-modifies cell lysates, but Ac_4_2AzMan labels proteins in living cells. Native **(A)** or denatured **(B)** HeLa cell lysates or living HeLa cells **(B)** were treated with the indicated concentrations of the MCRs at 37°C for 2 or 16 h, respectively. After this length of time, CuAAC with alkyne-TAMRA was performed and any labeled proteins were visualized by in-gel fluorescence scanning.

In order to further explore the potential of structural diverse monosaccharide analogs as potential MCRs, we purchased Ac_4_2AzMan and synthesized Ac_4_4AzGal in a two-step, one-pot synthesis from commercially available 1,2,3,6-*O*-acetyl-glucose (Supporting Information). We then incubated these compounds (200 μM or 2 mM) at 37°C for 2 h with HeLa cell lysates, under the same conditions previously reported to result in chemical modification of cysteine residues (Qin et al., 2018), as well as chemically-denatured (1% SDS) cell lysates. In parallel, we treated HeLa cells in culture with the same compounds (200 μM) for 16 h, our standard MCR labeling protocol. The samples were then subjected to copper(I)-catalyzed azide-alkyne cycloaddition (CuAAC) with alkyne-TAMRA and then analyzed by in-gel fluorescence (Figure 2). Incubation of the reporters with cell lysates resulted in a small amount of labeling over background that was essentially identical for the two compounds. However, we observed noticeably higher live-cell labeling for Ac_4_2AzMan compared to Ac_4_4AzGal. Notably, the pattern of both reporter-modified proteins in lysates largely matches those proteins that non-selectively react with alkyne-TAMRA during CuAAC, presumably abundant proteins in the lysate. This pattern is conserved in the cells labeled with Ac_4_4AzGal, indicating that these proteins are indeed the result of background chemical modification. In contrast, cells treated with Ac_4_2AzMan resulted in the visualization of several unique protein bands. These results confirm that high concentrations of per-*O*-acetylated MCRs can result in at least low levels of background protein labeling, but also suggest that cellular metabolism is critical for the robust labeling observed with Ac_4_2AzMan treatment.

**Figure 3:**
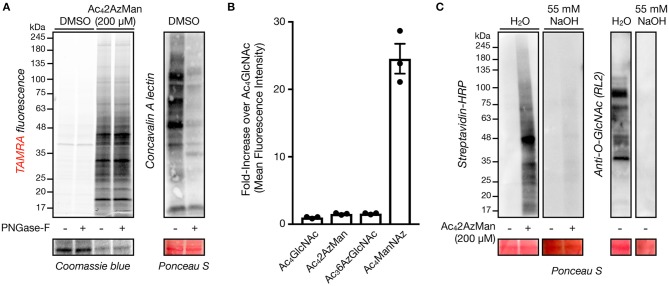
Ac_4_2AzMan labels intracellular proteins and is likely on serine, threonine, and/or cysteine residues. **(A)** Removal of N-linked glycans has no effect on Ac_4_2AzMan labeling. H1299 cells were treated with Ac_4_2AzMan (200 μM) or vehicle for 16 h before treatment of the corresponding lysates with PNGase-F to remove N-linked glycans. Any protein labeling was then observed by in-gel fluorescence scanning after CuAAC with alkyne-TAMRA. Lectin blotting with concavalin A confirmed the removal of N-linked glycans. **(B)** Ac_4_2AzMan-treatment does not result in cell-surface labeling. NIH3T3 cells were incubated with the indicated MCRs (200 μM) for 16 h. The cells were then harvested, reacted with DBCO-biotin, incubated with FITC-streptavidin, and analyzed by flow cytometry. Error bars represent ±s.e.m. from the mean of three biological replicates (*n* = 3). **(C)** β-Elimination results in loss of Ac42AzMan labeling. H1299 cells were treated with Ac42AzMan (200 μM) or vehicle for 16 h. After this time, the corresponding lysates were subjected to CuAAC with alkyne-biotin and SDS-PAGE. After transfer the corresponding PVDF membranes were incubated with either NaOH or H_2_O before streptavidin or Western blotting.

We next set out to characterize the labeling of living cells by Ac_4_2AzMan. First, we explored the possibility that the reporter is incorporated into cell-surface glycosylation. Accordingly, H1299 cells were treated with Ac_4_2AzMan (200 μM) or vehicle for 16 h. We chose this concentration, as in our experience acetylated MCRs are often toxic to cells at higher concentrations. The corresponding total cell lysates were then split and treated with water or PNGase-F for 5 h at 37°C to enzymatically remove N-linked glycosylation. The samples were then subjected to CuAAC with alkyne-TAMRA and analyzed by in-gel fluorescence and lectin blotting (Figure 3A). The fluorescent gel scanning showed no loss of signaling upon PNGase-F treatment but dramatic removal of N-linked glycans as visualized by Concanavalin A (ConA), demonstrating that the MCR is not incorporated into this type of glycosylation to any significant extent. Next, we used flow cytometry to more broadly examine the potential incorporation of Ac_4_2AzMan into cell-surface glycoconjugates. More specifically, NIH3T3 cells were treated with Ac_4_2AzMan (200 μM) or vehicle for 16 h. Simultaneously, the same cell-line was treated separately with either Ac_4_ManNAz (200 μM) or Ac_3_6AzGlcNAc (200 μM). Ac_4_ManNAz treatment serves as a positive control for cell surface labeling (Saxon and Bertozzi, 2000), while we have previously shown that Ac_3_6AzGlcNAc treatment results in the exclusive modification of intracellular proteins (Chuh et al., 2014). After 16 h, the cells were released, reacted with DBCO-Biotin, and incubated with FITC-Avidin before analysis by flow-cytometry (Figure 3B). As expected, we observed high levels of labeling after Ac_4_ManNAz treatment but essentially no signal over background from Ac_3_6AzGlcNAc treated cells. Consistent with our PNGase-F experiment, we also found no cell-surface labeling with Ac_4_2AzMan. Next, we took advantage of β-elimination chemistry to test whether the observed signal was due to base labile modifications on residues such as serine, threonine, or cysteine. H1299 cells were first treated with Ac_4_2AzMan (200 μM) or vehicle for 16 h before the corresponding cell lysates were subjected to CuAAC with alkyne-biotin. The samples were then ran in duplicate on SDS-PAGE and transferred to a nitrocellulose membrane. The membranes were then incubated at 40°C for 24 h in either water or 55 mM NaOH and then visualized using streptavidin blotting (Figure 3C). We found that the β-elimination conditions removed essentially all of the Ac_4_2AzMan labeling. As a control for the chemistry, we also visualized the loss of intracellular O-GlcNAc modifications by Western blotting under the same conditions (Figure 3C).

**Figure 4:**
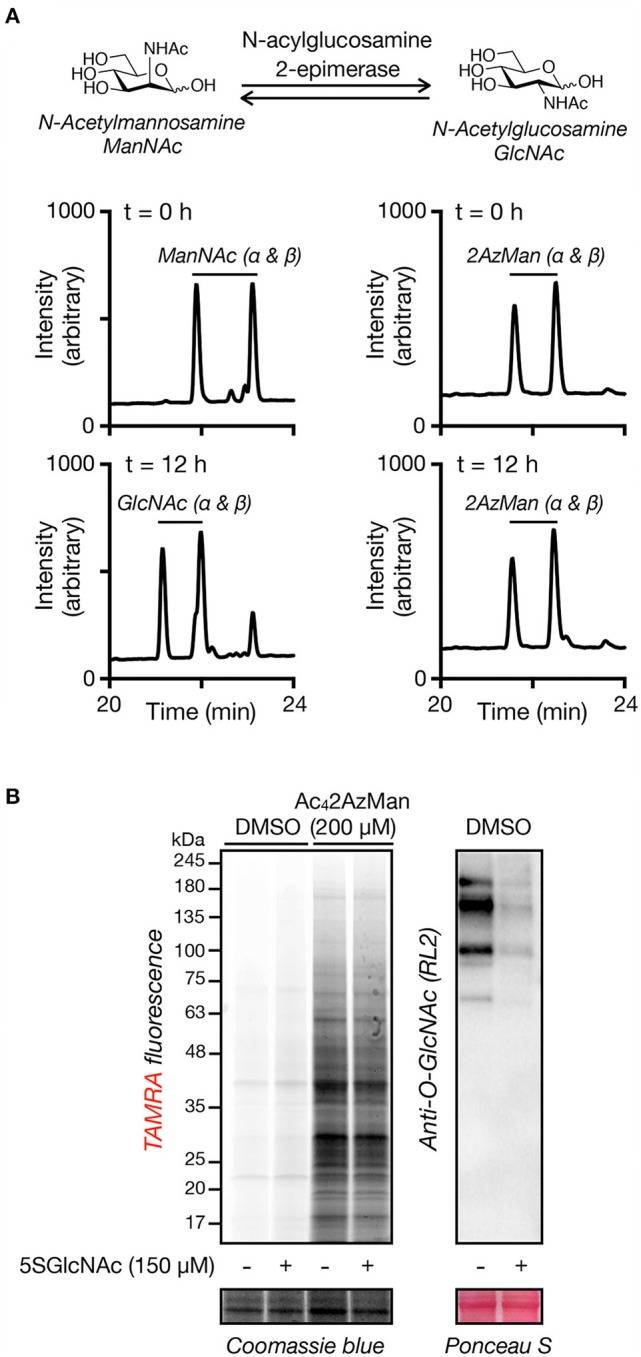
Ac_4_2AzMan labeling is not due to enzymatic epimerization to 2AzGlc or O-GlcNAc transferase activity. **(A)** 2AzMan is not enzymatically epimerized to 2AzGlc. ManNAc or 2AzMan was independently incubated with recombinant N-acylglucosamine 2-epimerase for 12 h before detection of the formation of any corresponding epimerization by HPLC. **(B)** OGT inhibition does not notably reduce 2AzMan labeling. H1299 cells were co-treated with Ac_4_2AzMan (200 μM) and either the OGT inhibitor Ac_4_5SGlcNAc (150 μM) or DMSO for 16 h before visualization of labeled proteins by CuAAC and in-gel fluorescence scanning.

The fact that Ac_4_2AzMan results in intracellular protein modification raised the possibility that it was entering the O-GlcNAc modification pathway. More specifically, we hypothesized that 2AzMan might be enzymatically converted to 2-azido-2-deoxy-glucose (2AzGlc) by the enzyme *N*-acetylglucosamine 2-epimerase, as we and the Vocadlo lab demonstrated that 2AzGlc is an MCR for O-GlcNAcylation (Shen et al., 2017; Zaro et al., 2017). To directly test this possibility, we first incubated an anomeric mixture of α- and β-ManNAc with either buffer or recombinantly expressed *N*-acylglucosamine 2-epimerase (Uniprot P51606) for 12 h before analysis by HPLC (Figure 4A). As expected, *N*-acetylmannosamine (ManNAc) was enzymatically converted to two new peaks consisting of the α- and β-anomers of GlcNAc. In contrast, we observed no conversion of 2AzMan to 2AzGlc, rejecting our conversion hypothesis (Figure 4A). Simultaneously, we also co-treated H1299 cells with Ac_4_2AzMan (200 μM) and either the OGT inhibitor Ac_4_5SGlcNAc (150 μM) or DMSO for 16 h (Gloster et al., 2011). The lysates were then subjected to CuAAC with alkyne-TAMRA and labeling visualized by in-gel fluorescence (Figure 4B). In support of our *in vitro* experiment, we observed very little loss of Ac_4_2AzMan labeling upon OGT inhibition. In contrast, a similar OGT inhibition experiment with Ac_4_2AzGlc previously resulted in loss of over half of the labeling (Shen et al., 2017). Together, these experiments argue against the conversion of 2AzMan to 2AzGlc and subsequent incorporation into O-GlcNAc modifications.

**Figure 5:**
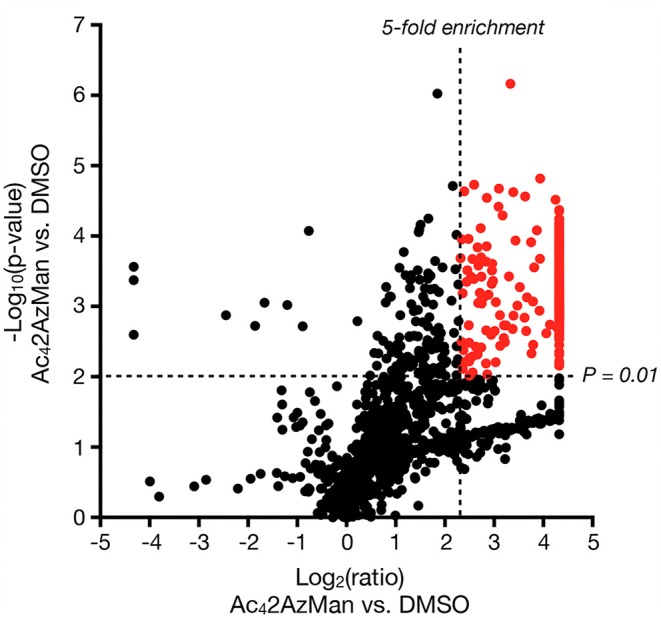
Ac_4_2AzMan labels a wide variety of intracellular proteins. H1299 cells were treated with either Ac_4_2AzMan (200 μM) or DMSO vehicle for 16 h. Labeled proteins were then enriched using neutravidin beads after CuAAC with alkyne-biotin. Proteins were then identified using label free quantitation after on-bead trypsinolysis and LC-MS/MS. The results are shown as a Volcano Plot (x-axis: log_2_ ratio of MCR to vehicle, y-axis; –log_10_ *p*-value). Significantly enriched proteins that differ at least 5 linear-fold with a *p* < 0.01 (Student's *t*-test) are marked in red.

Next, we set out to identify the 2AzMan-modified proteins. Accordingly, we treated H1299 cells in triplicate with either Ac_4_2AzMan (200 μM) or DMSO vehicle for 16 h, followed by CuAAC with alkyne-biotin and enrichment of any modified proteins with neutravidin beads. We then performed on-bead trypsinolysis and identification of the resulting peptides by Label Free Quantitative (LFQ) proteomics (Cox et al., 2014) (*n* = 3 biological replicates with a false discovery rate of 0.01). This allowed us to identify over 1,000 2AzMan-labeled proteins based on several criteria (Figure 5 and Table S1): the protein must have been identified in at least 2 out of the 3 biological replicates, the enrichment ratio (LFQ-based) must have been at least 5 linear-fold greater in the treated samples vs. vehicle, and the statistical significance (p-value) of this difference must have been <0.01 (Student's *t*-test) This cutoff was chosen arbitrarily and is fairly stringent; however, a full list of proteins enriched at lower ratios can be found in Table S1. Consistent with our other biochemical analysis, the enriched proteins represented a wide-range of intracellular proteins. We next employed the IsoTaG platform (Woo et al., 2015, 2017) to determine if 2AzMan or 4AzGal could be directly identified on proteins and the specific sites of those modifications. We again treated H1299 cells with either Ac_4_2AzMan (200 μM), Ac_4_4AzGal (200 μM), or DMSO vehicle in duplicate for 16 h. We then subjected the corresponding lysates to CuAAC with a mixture of isotopically-labeled, cleavable biotin tags. After enrichment of the labeled proteins on streptavidin beads and on-bead trypsinolysis, we eluted the directly modified, and therefore isotopically encoded, peptides using weak acid. Subsequent LC-MS analysis using the IsoStamp v2.0 software was then used to look for un-, mono-, di-, or tri-acetylated azido-hexose modification of any Asn, Ser, Thr, or Cys residue. Using IsoTaG, we were able to localize 2AzMan on 33 peptides, with all of the modifications on Cys (Table 1 and Table S2), while we found no peptides modified by 4AzGal. Overall our results are consistent with the background MCR labeling of proteins on cysteine residues seen by Chen and Wang (Qin et al., 2018; Hao et al., 2019) but also indicate that cellular metabolism of Ac_4_2AzMan plays an important role that distinguishes it from Ac_4_4AzGal.

**Table 1:** 2AzMan-modified peptides.

**Entry**	**Peptide (modification site bolded)**	**Protein name**	**Uniprot accession**
1	[K].AGQ**C**VIGLQMGTNK.[C]	Calponin-2	Q99439
2	[K].AIN**C**ATSGVVGLVNCLR.[R]	Fatty acid synthase	P49327
3	[K].AMSYKPICPT**C**QTSYGIQK.[G]	DTX3L	Q8TDB6
4	[K].APPPSLTD**C**IGTVDSR.[A]	Charged multivesicular body protein 5	Q9NZZ3
5	[K].AVQDL**C**GWR.[I]	Protein RCC2	Q9P258
6	[K].ETTNIFSNCG**C**VR.[A]	Ataxin-10	Q9UBB4
7	[K].EINYVKN**C**ER.[A]	Nucleophosmin	P06748
8	[K].HELQANCYEEVKDR**C**TLAEK.[L]	Cofilin-1	P23528
9	[K].HFLSDTGMA**C**R.[S]	5′-nucleotidase domain-containing protein 1	Q5TFE4
10	[K].NCG**C**LGASPNLEQLQEENLK.[L]	Arginine-tRNA ligase	P54136
11	[K].NSNVDSSYLESLYQS**C**PR.[G]	Zinc finger CCCH-type antiviral protein	Q7Z2W4
12	[K].NVE**C**AR.[V]	Charge multivesicular body protein 1a	Q9HD42
13	[K].SGIQPL**C**PER.[S]	Lamina-associated polypeptide 2	P42166
14	[K].SSG**C**DVNLPGVNVK.[L]	Neuroblast differentiation-associated protein	Q09666
15	[K].STACQMLV**C**YAK.[L]	Importin-5	O00410
16	[K].STLTDSLV**C**K.[A]	Elongation Factor 2	P13639
17	[K].STTTSDPPNI**C**K.[V]	Protein PRRC2C	Q9Y520
18	[K].TV**C**DAAEK.[G]	Perilipin-3	O60664
19	[K].TVGVQGD**C**R.[S]	GMP synthase	P49915
20	[K].VA**C**AEEWQESR.[T]	TIP41-liked protein	O75663
21	[K].VVNEINIEDL**C**LTK.[A]	CDGSH iron-sulfur domain-containing protein	Q8N5K1
22	[M].A**C**ARPLISVYSEK.[G]	60S ribosomal protein L4	P36578
23	[M].A**C**GLVASNLNLKPGECLR.[V]	Galectin-1	P09382
24	[R].EVGGGHG**C**TSPPFPQEAR.[A]	Portein lin-28 homolog B	Q6ZN17
25	[R].FCEMCCD**C**R.[M]	Serine/threonine-protein phosphatase 4 regulatory subunit 1	Q8TF05
26	[R].IFGSIPMQA**C**QQK.[D]	Zinc finger CCCH domain-containing protein 8	Q6NZY4
27	[R].INISEGNB**C**PER.[I]	Poly(rC)-binding protein 2	Q15366
28	[R].LQGIN**C**GPDFTPSFANLGR.[T]	Eukaryotic translation initiation factor 4	Q04637
29	[R].LVVPATQ**C**GSLIGK.[G]	Poly(rC)-binding protein 1	Q15365
30	[R].LYYFQYP**C**YQEGLR.[S]	APOBEC-3C	Q9NRW3
31	[R].NLFIS**C**K.[S]	Lamina-associated polypeptide 2, isoform alpha	P42166
32	[R].SILSPGGS**C**GPIK.[V]	General transcription factor II	P78347
33	[R].VMTIPYQPMPASSPVI**C**AGGQDR.[C]	Poly(rC)-binding protein 1	Q15365

**Figure 6:**
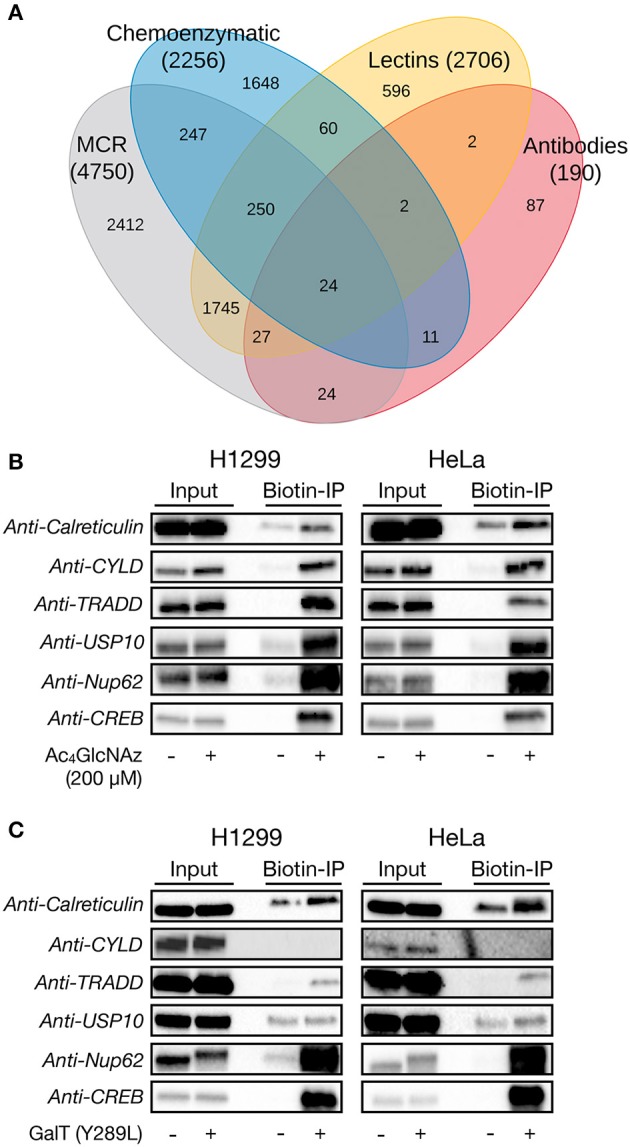
Per-*O*-acetylated MCRs are imperfect but reasonable tools for the identification of potentially O-GlcNAcylated proteins. **(A)** Venn diagram showing the overlap between proteins that have been identified as being potentially O-GlcNAcylated by different enrichment methods. **(B)** H1299 or HeLa cells were treated with the widely-used O-GlcNAc MCR Ac_4_GlcNAz (200 μM) for 16 h, followed by CuAAC with a cleavable biotin linker, enrichment, release, and analysis by Western blotting. **(C)** H1299 or HeLa lysates were subjected to chemoenzymatic labeling of endogenous O-GlcNAc modifications with the same cleavable biotin linker. Modified proteins were visualized by Western blotting after enrichment and release.

The chemical modification of intracellular proteins upon treatment of living cells with certain MCRs could detrimentally affect the discovery of legitimately O-GlcNAcylated proteins. To explore this question, we performed a meta-analysis of potential O-GlcNAcylated proteins identified using common MCRs for O-GlcNAc (Ac_4_GlcNAz, Ac_4_GalNAz, Ac_3_6AzGlcNAc, etc.) (Worth et al., 2017) and with other methods that detect endogenous O-GlcNAcylation (lectin chromatography, anti-O-GlcNAc antibodies, or chemoenzymatic modification). A complete list of the proteomics studies used in this analysis is available in the Supporting Information. More specifically, we used python scripts (Scripts S1–S3). The first script produces one comprehensive file containing all of the identified proteins and their associated proteomic studies, ordered by the occurrences for each protein from most to least, which should be of general interest to the field and allow for the easy identification of potentially O-GlcNAcylated proteins across datasets. The second and third scripts first return a list a proteins identified by a particular method (e.g., MCR treatment) and then generate the numbers of exclusive and overlapping proteins for each identification technique, allowing us to generate a large Venn diagram (Figure 6A). Consistent with a significant amount of published work using MCRs to discover new O-GlcNAc modified proteins, essentially half of the MCR-identified proteins were also found to be O-GlcNAcylated by at least one of the other techniques. However, the MCRs also yielded the highest number of exclusively identified proteins. We reasoned that these proteins could arise from the chemical modification of proteins, the induction of O-GlcNAcylation by MCR treatment, or reporting on endogenous glycosylation that was missed during the proteomic analysis using other techniques. To estimate how many of the proteins exclusive to MCRs fall into this last category of bonafide O-GlcNAcylated proteins, we selected four such proteins at random: TRADD (Uniprot Q15628), calreticulin (Uniprot P27797), USP10 (Uniprot Q14694), and CYLD (Uniprot Q9NQC7). We then treated H1299 or HeLa cells with Ac_4_GlcNAz (200 μM) for 16 h, followed by CuAAC with a cleavable biotin-linker (Darabedian and Pratt, 2019). The modified proteins were then enriched on streptavidin beads, extensively washed, and eluted before visualization by Western blotting (Figure 6B). As expected from the proteomic data, we found all four of these proteins to be enriched, as well as the known O-GlcNAcylated proteins Nup62 and CREB. Simultaneously, we subjected H1299 and HeLa cell lysates to chemoenzymatic modification followed by the same CuAAC and enrichment procedure. Analysis by Western blotting showed enrichment over background of TRADD and calreticulin in both cells lines, confirming their O-GlcNAcylation status, while CYLD was not enriched in either cell line (Figure 6C). These data suggest that the commonly used MCRs for O-GlcNAc probably do result in the enrichment and false identification of some proteins that are not endogenously modified. However, they also indicate that this number is not overwhelmingly large, with a crude estimation that of the proteins only identified using an MCR ~50% are real O-GlcNAcylated proteins that can be confirmed using another technique. Combining this estimation with the documented overlap with other techniques in the Venn diagram suggests that around 75% of the proteins found by MCRs are indeed modified.

## Discussion

The relatively recent discovery that per-*O*-acetylated MCRs can label cysteine residues when incubated this protein lysates (Qin et al., 2018; Hao et al., 2019) has raised important questions about some of the biological conclusions that have been drawn using these tools. This is particularly true for MCRs that target intracellular O-GlcNAcylation due to the increased abundance of free cysteine sulfhydryl groups compared to the cell surface, where many cysteines are found as oxidized disulfides. Despite good evidence for this background modification, the proposed mechanism of a reaction between cysteine sides-chains and the anomeric *O*-acetate (Qin et al., 2018) of the MCR was somewhat chemically unsatisfying.

Here, we demonstrate that at least some background, chemical labeling of proteins is due to selective metabolism of certain MCRs by living cells. Specifically, when we incubated cell lysates with two potential MCRs, Ac_4_2AzMan, and Ac_4_4AzGal (Figure 1B), we observed essentially no labeling (Figure 2). However, Ac_4_2AzMan showed robust labeling of protein in living cells, while Ac_4_4AzGal did not (Figure 2). Using a variety of biochemical techniques, we demonstrated that Ac_4_2AzMan labeling is not due to incorporation into cell surface glycosylation (Figures 3A,B) but is instead found on various intracellular proteins, most likely through serine, threonine, or cysteine residues (Figure 3C). This raised the possibility that 2AzMan is enzymatically epimerized into the previously characterized O-GlcNAc MCR, 2AzGlc (Shen et al., 2017; Zaro et al., 2017). However, we ruled out this possibility using both *in vitro* enzymology (Figure 4A) and in cells through inhibition of O-GlcNAc transferase (Figure 4B). We then confirmed our biochemical analysis using proteomics to show widespread labeling of intracellular proteins (Figure 5 and Table S1) and essentially exclusive modification of cysteine residues over other potential side chains (Table 1 and Table S2). Together, our data support the work by Wang and Chen by demonstrating that background modification of proteins by *O*-acetylated MCRs is certainly a possibility. However, we also show that this labeling is not exclusively because of direct chemical modification of proteins by per-O-acetylated MCRs but can also result from metabolism in living cells. Notably, this metabolism-dependent, background labeling is not universal, since Ac_4_4AzGal treatment does not result in protein modification.

We do not know yet know the metabolic pathways that are involved in this observation, but we speculate that it could be the deacetylation of different hydroxyl groups on the MCR. In particular, the enzymatic deacetylation of the 1-hydroxyl of any monosaccharide could result in the generation of reactive aldehyde. Importantly, this is consistent with the observation that MCRs with a free anomeric position more readily react with proteins (Hao et al., 2019) and display increased cellular toxicity (Aich et al., 2008). The fact that we detected partially *O*-acetylated-2AzMan on cysteines in the proteomics data supports this possibility as at least contributing to the labeling. It is equally possible that inherent differences in the chemical structure and therefore reactivity of the MCRs is the driving force behind the different levels of cellular labeling. For example, after deacetylation the azide at the 2-position of 2AzMan would result in a very stereoelectronically different environment around the reactive 1-aldehyde compared to 4AzGal. Therefore, it is also possible that the MCRs are metabolized similarly by the cells but then modify proteins because of reactivity differences derived from their chemical structures. It is also important to point out that we do not believe that Ac_4_2AzMan is acting as a reporter for glycosyltransferase-mediated labeling of cysteine residues but rather as a precursor for a reactive metabolite that results in their chemical modification. Finally, our results strongly support the use of glycosite mapping compared to simply protein identification in proteomics. At the glycosite level, MCR-modification of serines and threonines, which are likely enzymatic modifications, can easily be distinguished from cysteine modifications that may be background. In fact, the numerous O- and N-linked glycan modification sites that have been identified using MCRs are almost certainly due to enzymatic addition, further highlighting the utility of metabolic probes.

Together, these results suggest that many of the proteins that have been identified as being O-GlcNAcylated by MCRs may be background-labeled proteins instead. To investigate this possibility, we performed an analysis of proteins who had been previously identified as being potentially O-GlcNAcylation using different techniques, including MCRs, chemoenzymatic modification, or lectin- or antibody-based enrichment (Figure 6A). Notably, we found that MCR-based identification did not result in an inordinate amount of unique identifications compared with the other techniques, all of which enrich endogenous O-GlcNAc modifications. However, given the potential for “off-target” labeling by MCRs and the largest number of potential O-GlcNAcylated proteins uniquely identified using these tools, we randomly chose 4 proteins from the “MCR-unique” list and first confirmed that a common O-GlcNAc-targeted MCR would indeed enrich these proteins (Figure 6A). We then used chemoenzymatic enrichment to determine if we could confirm that these proteins are indeed O-GlcNAcylated and found that at least 2 of them are endogenously modified (Figure 6B). In summary, our results further confirm that per-O-acetylated monosaccharide MCRs can label proteins in a way that does not necessarily reflect their glycosylation status. Despite this, we also found that overall MCRs are fairly reliable tools for the identification of O-GlcNAcylated proteins and should not be discarded but instead complementary methods should simply be used to confirm any potentially modified proteins.

## Data Availability Statement

The datasets generated for this study can be found in the ProteomeXchange Consortium under accession number PXD016217. Additionally, publicly available datasets were analyzed in this study. This data can be found in UniProt under the accession numbers listed in Table 1.

## Author Contributions

ND, BY, RD, GC, BZ, CW, and MP designed experiments and interpreted data. ND carried out the synthesis of MCRs. ND and GC performed analysis of MCR labeling in cell lysates. ND performed the cellular analysis of MCR labeling, flow cytometry, β-elimination, analysis of potential MCR epimerization, and MCR/chemoenzymatic IP analyses. BZ performed protein-level proteomic analysis. BY performed site-identification of MCR labeling by proteomics. RD generated the Python scripts and performed meta-analysis. ND, BY, BZ, CW, and MP prepared the manuscript.

## Conflict of Interest

The authors declare that the research was conducted in the absence of any commercial or financial relationships that could be construed as a potential conflict of interest.

## Supplementary Material

Synthetic methods for Ac_4_4AzGal preparation, NMR characterization of Ac_4_4AzGal, list of published proteomic results used for meta-analysis in Figure 6A, Python scripts used to generate Figure 6A, instructions for the use of these Python scripts, proteomics data for both protein- (Table S1) and site-identification (Table S2).
