# Supplementary material for: O-Acetylated Chemical Reporters of Glycosylation Can Display Metabolism-Dependent Background Labeling of Proteins but Are Generally Reliable Tools for the Identification of Glycoproteins
Source: Front Chem. 2020 Apr 28;8:318. doi: 10.3389/fchem.2020.00318 (PMC7198827; doi:10.3389/fchem.2020.00318)
Supplement: Supplementary file 3 [file Data_Sheet_1.PDF]

O-Acetylated chemical reporters of glycosylation can display metabolism-dependent background labeling of proteins but are generally reliable tools for the identification of glycoproteins

Narek Darabedian[1], Bo Yang,[2] Richie Ding,[4] Giuliano Cutolo,[1] Balyn W. Zaro,[3] Christina M. Woo,[2] and Matthew R. Pratt[1,4*]
[1]Departments of Chemistry and [4]Biological Sciences, University of Southern California, Los Angeles, California, 90089
[2]Department of Chemistry and Chemical Biology, Harvard University, Cambridge, Massachusetts, 02138
[3]Department of Pharmaceutical Chemistry and The Cardiovascular Research Institute, University of California San Francisco, San Francisco, California, 94158
*Corresponding Author: Matthew R. Pratt
Email: matthew.pratt@usc.edu

Table of contents:

Synthesis of $Ac_4$4AzGal **Page S2**
NMR characterization of $Ac_4$4AzGal **Page S2**
Proteomic lists used for meta-analysis **Page S3**
Instructions for using Scripts S1 and S2 **Page S4**

References **Page S5**

Synthesis of known $Ac_4$4AzGal. (Lichtenthaler and Heidel, 1974) 1,2,3,6-Tetra-O-acetyl-a-D-glucopyranose (250 mg, 0.72 mmol) was dissolved in 7 mL DCM and 1 mL of pyridine was added and cooled to 0 °C. Then Triflic anhydride (0.49 mL, 2.87 mmol) was added dropwise and allowed to stir at 0 °C for 1 hour. The reaction was then diluted with DCM and washed twice with 1 M hydrogen chloride, saturated sodium bicarbonate, brine, dried over sodium sulfate and concentrated down. The resulting slurry wad dissolved in 5 mL DMF and NaN_3 was added and stirred for 1.5 hours. The reaction was then diluted with EtOAc and the organic layer was washed twice with water, brine, dried over sodium sulfate and was purified by column chromatography (10 to 30% acetone in hexanes, 3 steps) to afford 205 mg of the product. ^{1}H NMR (400 MHz, Chloroform-*d*) δ 6.31 (t, *J* = 1.6 Hz, 1H), 5.41 – 5.39 (m, 2H), 4.24 – 4.16 (m, 4H), 2.15 (s, 3H), 2.14 (s, 3H), 2.09 (s, 3H), 2.03 (s, 3H).

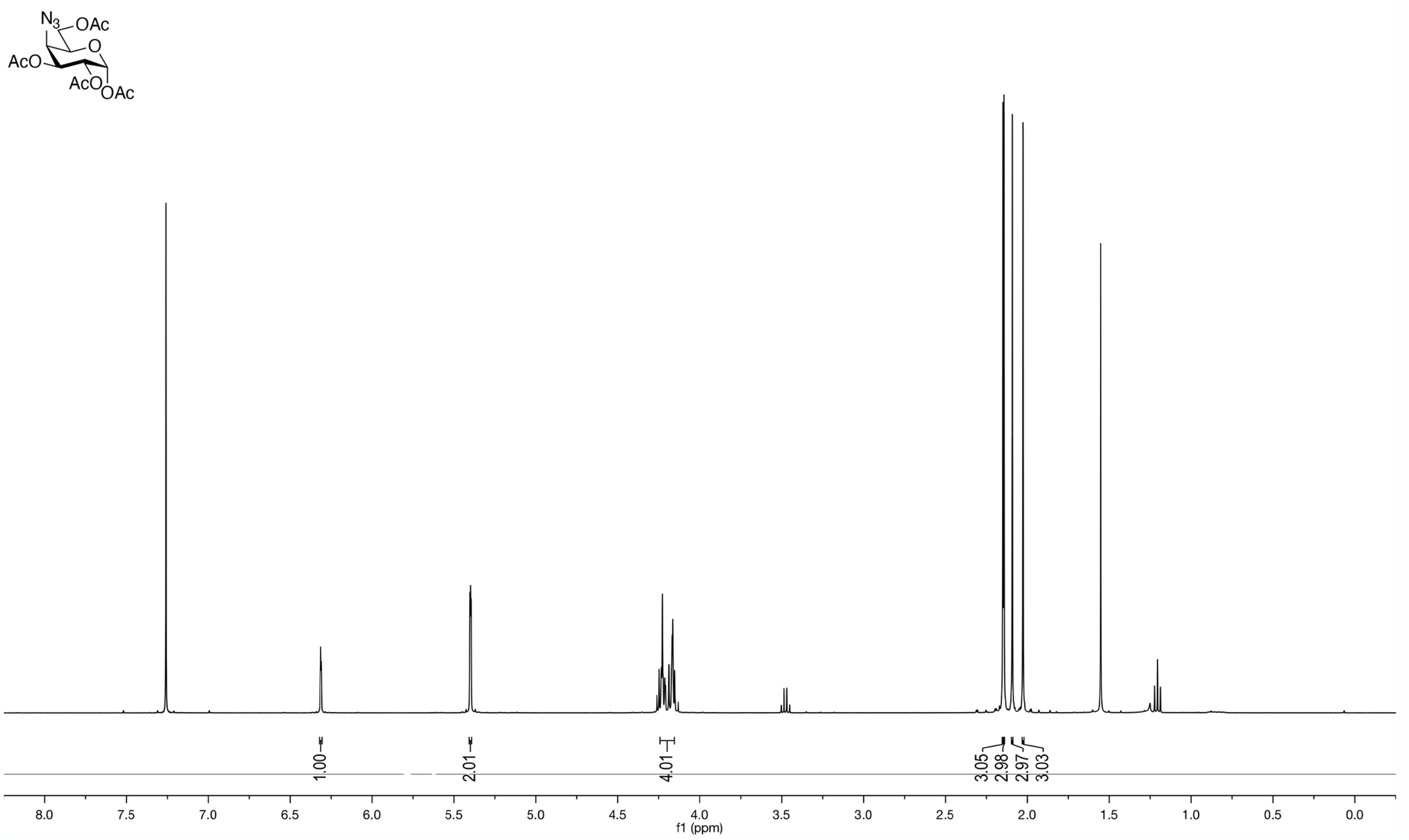

^{1}H NMR $Ac_4$4AzGal.

Proteomic lists used for meta-analysis

Lectins

Trinidad JC, Barkan DT, Gulledge BF, Thalhammer A, Sali A, Schoepfer R, et al. Global identification and characterization of both O-GlcNAcylation and phosphorylation at the murine synapse. Mol Cell Proteomics. 2012 Aug;11(8):215–29.

Morris M, Knudsen GM, Maeda S, Trinidad JC, Ioanoviciu A, Burlingame AL, et al. Tau post-translational modifications in wild-type and human amyloid precursor protein transgenic mice. Nat Neurosci. 2015 Aug;18(8):1183–9.

Antibodies

Wang Z, Pandey A, Hart GW. Dynamic Interplay between O-Linked N-Acetylglucosaminylation and Glycogen Synthase Kinase-3-dependent Phosphorylation. Mol Cell Proteomics. 2007 May 17;6(8):1365–79.

Teo CF, Ingale S, Wolfert MA, Elsayed GA, Nöt LG, Chatham JC, et al. Glycopeptide-specific monoclonal antibodies suggest new roles for O-GlcNAc. Nat Chem Biol. 2010 May 1;6(5):338–43.

Chemoenzymatic

Clark PM, Dweck JF, Mason DE, Hart CR, Buck SB, Peters EC, et al. Direct In-Gel Fluorescence Detection and Cellular Imaging of O-GlcNAc-Modified Proteins. J Am Chem Soc. 2008 Sep 3;130(35):11576–7.

Wang Z, Udeshi ND, O'Malley M, Shabanowitz J, Hunt DF, Hart GW. Enrichment and Site Mapping of O-Linked N-Acetylglucosamine by a Combination of Chemical/Enzymatic Tagging, Photochemical Cleavage, and Electron Transfer Dissociation Mass Spectrometry. Mol Cell Proteomics. 2010 Jan 6;9(1):153–60.

Wang Z, Udeshi ND, Slawson C, Compton PD, Sakabe K, Cheung WD, et al. Extensive Crosstalk Between O-GlcNAcylation and Phosphorylation Regulates Cytokinesis. Science Signaling. AAAS; 2010 Jan 12;3(104):ra2.

Alfaro JF, Gong C-X, Monroe ME, Aldrich JT, Clauss TRW, Purvine SO, et al. Tandem mass spectrometry identifies many mouse brain O-GlcNAcylated proteins including EGF domain-specific O-GlcNAc transferase targets. Proc Natl Acad Sci USA. 2012 Apr 19;109(19):7280–5.

Wang S, Yang F, Petyuk VA, Shukla AK, Monroe ME, Gritsenko MA, et al. Quantitative proteomics identifies altered O-GlcNAcylation of structural, synaptic and memory-associated proteins in Alzheimer's disease. J Pathol. 2017 Sep;243(1):78–88

Qin K, Zhu Y, Qin W, Gao J, Shao X, Wang Y-L, et al. Quantitative Profiling of Protein O-GlcNAcylation Sites by an Isotope-Tagged Cleavable Linker. ACS Chem Biol. 2018 Aug 17;13(8):1983–9.

Li J, Li Z, Duan X, Qin K, Dang L, Sun S, et al. An Isotope-Coded Photocleavable Probe for Quantitative Profiling of Protein O-GlcNAcylation. ACS Chem Biol. 2019 Jan 18;14(1):4–10.

MCRs

Nandi A, Sprung R, Barma DK, Zhao Y, Kim SC, Falck JR, et al. Global identification of O-GlcNAc-modified proteins. Anal Chem. 2006 Jan 15;78(2):452–8.

Zaro BW, Yang Y-Y, Hang HC, Pratt MR. Chemical reporters for fluorescent detection and identification of O-GlcNAc-modified proteins reveal glycosylation of the ubiquitin ligase NEDD4-1. Proc Natl Acad Sci USA. 2011 May 3;108(20):8146–51.

Hahne H, Sobotzki N, Nyberg T, Helm D, Borodkin VS, van Aalten DMF, et al. Proteome Wide Purification and Identification of O-GlcNAc-Modified Proteins Using Click Chemistry and Mass Spectrometry. J Proteome Res. 2013 Feb;12(2):927–36.

Chuh KN, Batt AR, Zaro BW, Darabedian N, Marotta NP, Brennan CK, et al. The New Chemical Reporter 6-Alkynyl-6-deoxy-GlcNAc Reveals O-GlcNAc Modification of the Apoptotic Caspases That Can Block the Cleavage/Activation of Caspase-8. J Am Chem Soc. 2017 Jun 14;139(23):7872–85.

Qin W, Lv P, Fan X, Quan B, Zhu Y, Qin K, et al. Quantitative time-resolved chemoproteomics reveals that stable O-GlcNAc regulates box C/D snoRNP biogenesis. Proc Natl Acad Sci USA. 2017 Aug 15;114(33):E6749–58.

Woo CM, Felix A, Byrd WE, Zuegel DK, Ishihara M, Azadi P, et al. Development of IsoTaG, a Chemical Glycoproteomics Technique for Profiling Intact N- and O-Glycopeptides from Whole Cell Proteomes. J Proteome Res. 2017 Mar 7;16(4):1706–18.

Woo CM, Lund PJ, Huang AC, Davis MM, Bertozzi CR, Pitteri SJ. Mapping and Quantification of Over 2000 O-linked Glycopeptides in Activated Human T Cells with Isotope-Targeted Glycoproteomics (Isotag). Mol Cell Proteomics. 2018 Apr;17(4):764–75.

Qin K, Zhu Y, Qin W, Gao J, Shao X, Wang Y-L, et al. Quantitative Profiling of Protein O-GlcNAcylation Sites by an Isotope-Tagged Cleavable Linker. ACS Chem Biol. 2018 Aug 17;13(8):1983–9.

Instructions for using Scripts S1 and S2

An initial python script was used to consolidate several proteomics data files together to produce one comprehensive file of proteins and the files they appear in, ordered by number of occurrences for each protein across all files from most to least. The script takes in an array of excel files as the input. As each file is traversed, two dictionaries are constructed. The first has the entries as the keys and the number of occurrences for each entry across all files as the values. The second dictionary has the entries as the keys and sets of filenames where each entry occurs as the values. Both dictionaries are outputted to a file in the format of each line being entry name followed by the filenames where this entry appears, in reverse sorted order of number of occurrences.

A second and third python script was used to find the intersection between different methods, MCR, Chemo, Lectin and Antibody. The second script is a basic script where it takes in an array of excel files, stores the entries in every file in a set, and returns an output file of the list of proteins of a particular method. The third script takes in four files produced by the second script, which each contain a list of proteins, and finds the intersection as well as the mutually exclusive parts of the lists. The output file is ordered as 1, 2, 3, 4, 12, 13, 14, 123, 124, 134, 23, 24, 234, 34. Using these lists, we can produce a Venn diagram for all four methods.

Protein.py:

1. Navigate to location of the script
2. run script with 2 input parameters
    a. first parameter is option, 1 for protein ID, 2 for gene name
    b. second parameter is the actual files, enter an array of excel files
3. the output would be a text file named protein_output.txt

Sample use:
In Mac OS Terminal
python protein.py 1 ~/Desktop/protein_list/*.xlsx

site_list.py:

1. Navigate to location of the script
2. run script with 1 input parameters
    a. parameter is the actual files, enter an array of excel files

3. the output would be a text file named output.txt
4. needs to be done for each group (method) of lists

Sample use:
In Mac OS Terminal
python site_list.py ~/Desktop/protein_list/mcr/*.xlsx

intersection.py:
1. Navigate to location of the script
2. rename files produced from site_list.py to 1.txt, 2.txt, etc. (up to 4.txt)
3. run script
4. the output would be a text file named output.txt

Sample Use
In Mac OS Terminal
python intersection.py

References

Lichtenthaler, F. W., and Heidel, P. (1974). Preparative routes to 4-amino-4-deoxy-D-galactose. *The Journal of Organic Chemistry* 39, 1457–1462. doi:10.1021/jo00924a002.
